# Spatial Patterns of Plant Diversity, Biomass Allocation and Vegetation Biomass Carbon Storage in Major Mangrove Forests of Hainan, China

**DOI:** 10.3390/plants15152355

**Published:** 2026-07-30

**Authors:** Yuting Cao, Wei Zhou, Yuanying Peng, Siyao Liu, Junjie Lei, Wende Yan, Xiaoyong Chen

**Affiliations:** 1 Yuelushan Laboratory, Central South University of Forestry and Technology, Changsha 410004, China; rainy4ever@163.com; 2 School of Cultural and Creative Industries, Changsha Commerce & Tourism College, Changsha 410116, China; 3 Wildlife Division, Central South Inventory and Planning Institute of National Forestry and Grassland Administration, Changsha 410014, China; wei.seraph@163.com (W.Z.); liusiyao7608@163.com (S.L.); 4 College of Arts and Sciences, Lewis University, Romeoville, IL 60446, USA; pengyu@lewisu.edu; 5 College of Water and Soil Conservation, Central South University of Forestry and Technology, Changsha 410004, China; lailxy1314@126.com; 6 National Engineering Laboratory for Applied Technology of Forestry & Ecology in South China, Changsha 410004, China; 7 College of Arts and Sciences, Governors State University, University Park, IL 60484, USA

**Keywords:** species diversity, community structure, biomass allocation, carbon storage, mangrove ecosystems, tropical coastal wetlands

## Abstract

Mangrove ecosystems are among the most productive and carbon-dense coastal forests globally, playing a critical role in plant diversity conservation and climate regulation. However, spatial variability in plant diversity, biomass allocation, and vegetation biomass carbon storage remains insufficiently quantified at regional scales, particularly in biogeographically complex regions such as Hainan Island, China. Here, we investigated four representative mangrove regions in Hainan Province: Dongzhai Port (DPMD), Qinglan Port (QPMD), Yalong Bay (YBMD), and Xinying Port (XPMD). Based on 73 field plots (0.5 ha each), we quantified species composition, community diversity, importance value index (IVI), organ-level biomass allocation (leaves, stems, and roots), and vegetation biomass carbon storage using species-specific allometric equations and published carbon concentration factors. A total of 12 mangrove species from 7 families were recorded, with *Rhizophora stylosa* Griff. consistently dominating across all sites (IVI range: 19.8–58.7%). Significant spatial heterogeneity was observed in all measured parameters. QPMD exhibited the highest total biomass (92.2 t ha^−1^) and plant vegetation biomass carbon storage (40.1 t C ha^−1^), whereas YBMD showed the lowest biomass (26.1 t ha^−1^) and XPMD exhibited the strongest species dominance (Simpson’s D = 0.58). Stem biomass contributed the largest proportion (60–80%) across all sites, while root and leaf allocation varied significantly among regions, reflecting differences in community structure and stand characteristics. Vegetation biomass carbon storage patterns closely followed biomass distribution, demonstrating the dominant role of vegetation productivity in determining plant carbon accumulation. Our results reveal substantial spatial variation in mangrove diversity, biomass production, and vegetation biomass carbon storage. This study provides essential baseline information on mangrove vegetation biomass carbon stocks for region-specific conservation prioritization and climate mitigation strategies in tropical China.

## 1. Introduction

Mangrove wetlands are among the most productive and carbon-dense ecosystems on Earth, occupying a critical position at the land–sea interface in tropical and subtropical regions [1,2]. They provide essential services including shoreline stabilization, nutrient cycling, water purification, fisheries support and plant diversity conservation [3,4]. As ‘blue carbon’ ecosystems, mangroves store carbon at densities far exceeding most terrestrial forests due to high primary productivity and deep, organic-rich soils [5,6]. Although covering only 0.1% of the Earth’s surface [7], mangroves store up to five times more carbon per hectare than tropical rainforests, with global stocks estimated at 4.0–6.4 Pg C [5,8], and contribute 10–15% of global coastal carbon sequestration despite comprising less than 1% of tropical forest area [9,10]. Importantly, most carbon stored in mangrove ecosystems is contained within soil pools, while vegetation biomass represents an important aboveground and belowground carbon component that contributes to overall ecosystem carbon stocks [5]. However, this carbon storage capacity represents a vulnerability: degradation or conversion rapidly releases stored carbon, contributing substantially to global emissions [11,12], with mangrove loss accounting for approximately 10% of emissions from tropical deforestation [13].

Global assessments highlight both the ecological importance and vulnerability of mangroves. They cover approximately 137,760 km^2^ across 118 countries, with the Indo-Pacific region exhibiting the highest species diversity, containing over 40 of the world’s approximately 70 true mangrove species [14,15]. These ecosystems support complex food webs and provide habitat for numerous threatened species [16]. Intact mangrove ecosystems store substantial amounts of carbon, with an average ecosystem carbon stocks of approximately 1023 Mg C ha^−1^, of which 74% is stored in soils, highlighting the dominant contribution of belowground pools to total carbon storage [1,5]. However, despite their high ecological value, human activities, such as aquaculture, coastal land conversion, and timber extraction have caused a substantial global decline in mangrove cover over the past five decades [17,18]. Although the rate of loss has slowed, it remains at 0.2–0.7% per year in various regions [19]. Restoration efforts are increasing, but their success depends on spatially explicit ecological data on species composition, vegetation structure, and carbon and carbon stock dynamics to guide effective conservation and management strategies [20,21].

In China, mangrove forests have received increasing attention for climate change mitigation. Total mangrove area in China is approximately 33,000 ha, with ecosystem carbon storage estimated at 6.91 ± 0.57 Tg C [22]. Of this, 81.74% is stored in the top 1 m of soil, 18.12% in tree biomass, and 0.08% in litter [22], highlighting the importance of soil carbon pools [5]. Significant regional variation exists due to differences in climate, hydrology, and anthropogenic pressure [23,24]. Hainan Province, located in the tropical South China Sea (18°10′–20°10′ N), contains some of China’s most diverse and well-preserved mangroves. The island’s tropical climate (22–26 °C, 1500–2500 mm yr^−1^) and diverse coastal geomorphology support 25 true mangrove species, representing approximately 90% of China’s total mangrove diversity [25,26]. Major sites such as Dongzhai Port, Qinglan Port and Sanya Bay support distinct mangrove assemblages that are associated with variation in tidal regimes, geomorphology, freshwater inputs, soil characteristics, and disturbance histories [27,28]. Previous studies report substantial variability in vegetation biomass and carbon stocks across these sites, with plant carbon stocks varying among stands according to differences in species composition, forest structure, and environmental settings, while soil carbon represents the dominant long-term carbon pool in mature mangrove ecosystems [27,29,30].

Despite this recognized variability, comprehensive spatial assessments of biodiversity, biomass allocation, and plant carbon storage across Hainan’s mangroves remain limited. The relationships among species diversity, biomass production, and vegetation carbon stocks across different geomorphic settings have not been systematically quantified at the island scale [31,32]. Key knowledge gaps persist regarding how environmental gradients, and disturbance histories may be associated with variation in species distribution, community assembly and productivity. Furthermore, although the plant diversity–ecosystem functioning (BEF) hypothesis predicts positive relationships between plant diversity and ecosystem productivity, the extent to which plant diversity patterns are associated with biomass production, biomass allocation, and plant carbon storage remains insufficiently understood in mangrove systems [33,34]. Understanding these relationships is essential for predicting responses to environmental change and designing effective conservation and restoration interventions [35,36].

To address these gaps, this study provides a spatially explicit assessment of plant diversity, biomass production, and plant carbon storage across major mangrove ecosystems of Hainan Island. Specifically, we aimed to: (1) characterize species diversity, community structure, and importance values across four representative mangrove districts spanning the island’s northeastern, eastern, southern, and northwestern coasts, (2) quantify aboveground and belowground biomass production and allocation to leaves, stems, and roots using species-specific allometric equations, (3) identify spatial patterns in organ-level biomass ratios, and (4) estimate plant carbon storage and evaluate its relationship with spatial association with plant diversity metrics. We hypothesized that: (i) species diversity differs significantly among geomorphic settings due to environmental filtering, (ii) biomass production and allocation patterns vary significantly among districts in response to local hydrology, salinity, and disturbance histories (iii) spatial variation in species diversity is associated with variation in biomass production and plant carbon storage among mangrove regions, and (iv) plant carbon storage exhibits significant spatial variation and is positively associated with plant diversity metrics across mangrove ecosystems. These results provide essential baseline information for understanding spatial patterns of mangrove vegetation structure and plant carbon storage as a component of blue carbon ecosystems, supporting region-specific conservation and climate mitigation strategies in tropical coastal China [37].

## 2. Results

### 2.1. Species Composition, Importance Values, and Diversity

A total of 12 mangrove species from 7 families were recorded in the four study area (Table 1). The most species-rich families were Rhizophoraceae represented by five species: *Rhizophora stylosa* Griff., *Rhizophora apiculata* Blume, *Bruguiera gymnorrhiza* (L.) Lam., *Bruguiera sexangula* (Lour.) Poir., and *Kandelia obovata* Sheue, H.Y. Liu & J.W.H. Yong. The family Combretaceae included two species: Lumnitzera racemosa Willd. and Laguncularia racemosa (L.) C.F.Gaertn. Other recorded mangrove species included *Aegiceras corniculatum* (L.) Blanco, *Sonneratia apetala* Buch.-Ham., *Avicennia marina* (Forssk.) Vierh., *Hibiscus tiliaceus* L., and *Sonneratia caseolaris* (L.) Engl.

Across all regions, *R. stylosa* was the dominant species, exhibiting the highest IVI. This dominance reflected its high relative density, frequency, and basal area contribution within the mangrove communities. Other relatively important species included *S. apetala*, *K. obovata*, and *A. corniculatum*, which together with *R. stylosa* accounted for the majority of the total community importance. *L. racemosa* and *H. tiliaceus* showed moderate importance, whereas the remaining species contributed relatively less to the overall community structure (Table 2).

Region-level IVI analysis revealed substantial spatial variation in species importance, reflecting differences in local environmental conditions, disturbance histories, and community composition (Table 2). In DPMD, the community showed a relatively balanced structure, with *A. corniculatum*, *K. obovata*, and *R. stylosa* as the dominant species, while several other species contributed at lower levels. In contrast, QPMD was strongly dominated by *S. apetala* and *R. stylosa*, with *L. racemosa* as the only additional species recorded, resulting in the lowest species richness among the four districts. YBMD exhibited a relatively balanced co-dominance pattern among *R. stylosa, L. racemosa*, and *K. obovata*, with *A. marina* as a secondary component. XPMD showed the strongest dominance by *R. stylosa*, which had the highest single-species IVI among all districts, whereas *H. tiliaceus*, *L. racemosa*, and *R. apiculata* represented species of moderate importance.

**Figure 1:**
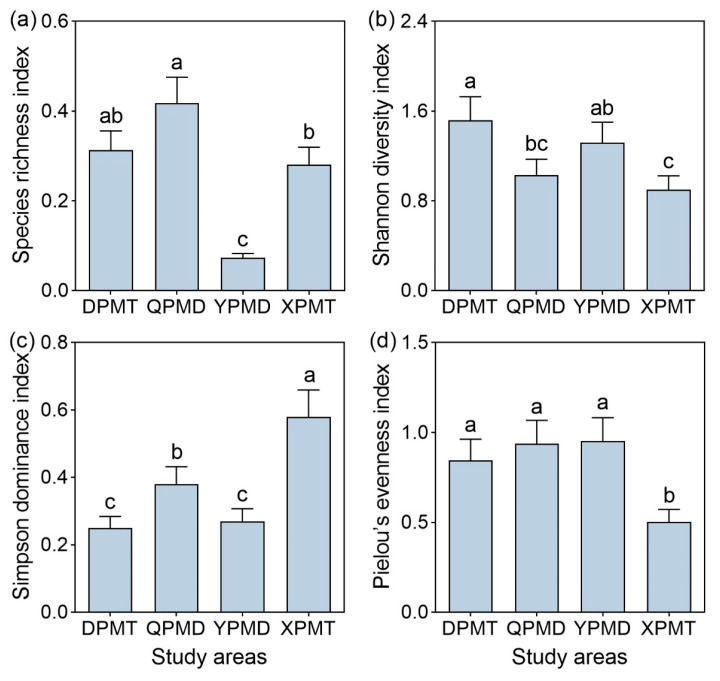
Variation in species diversity across the studied mangrove areas of Hainan. (**a**) Margalef species richness index (k), which adjusts species richness for sample size; (**b**) Shannon-Wiener diversity index (H′), reflecting both species richness and evenness; (**c**) Simpson’s dominance index (D), indicating the probability that two randomly selected individuals belong to the same species; and (**d**) Pielou’s evenness index (J), measuring the evenness of individual distribution across species. Different lowercase letters indicate significant differences among mangrove regions (*p* < 0.05), as determined by one-way ANOVA test followed by Tukey’s HSD test.

Species diversity varied significantly among the four mangrove districts (Figure 1). The Margalef richness index (k) was highest for QPMD and the average, whereas YBMD exhibited lowest value. The Shannon-Wiener diversity index (H′) showed significant variation (ANOVA: F_3,69_ = 8.74, *p* < 0.001). The overall average across all sampling plots (H′ = 1.70) and DPMD showed the highest diversity, YBMD showed intermediate diversity, while QPMD and XPMD exhibited the lowest diversity. Simpson’s dominance index (D) showed the inverse pattern, being greatest in XPMD, moderate in QPMD, and lowest in DPMD, YBMD, and the overall average across all sampling plots. Pielou’s evenness index (J) was highest in YBMD and the overall average across all sampling plots, and lowest in XPMD and QPMD and DPMD intermediate.

Fisher’s alpha diversity index also varied among mangrove districts. The highest Fisher’s alpha value was observed in DPMD (α = 1.235), followed by XPMD (α = 1.037), YBMD (α = 0.816), and QPMD (α = 0.502). Although XPMD had the highest number of individuals, its Fisher’s alpha diversity was relatively lower because of the strong dominance of *R. stylosa* in the community. In contrast, DPMD exhibited a more balanced species abundance distribution, resulting in the highest Fisher’s alpha value.

### 2.2. Biomass Production and Carbon Storage

**Figure 2:**
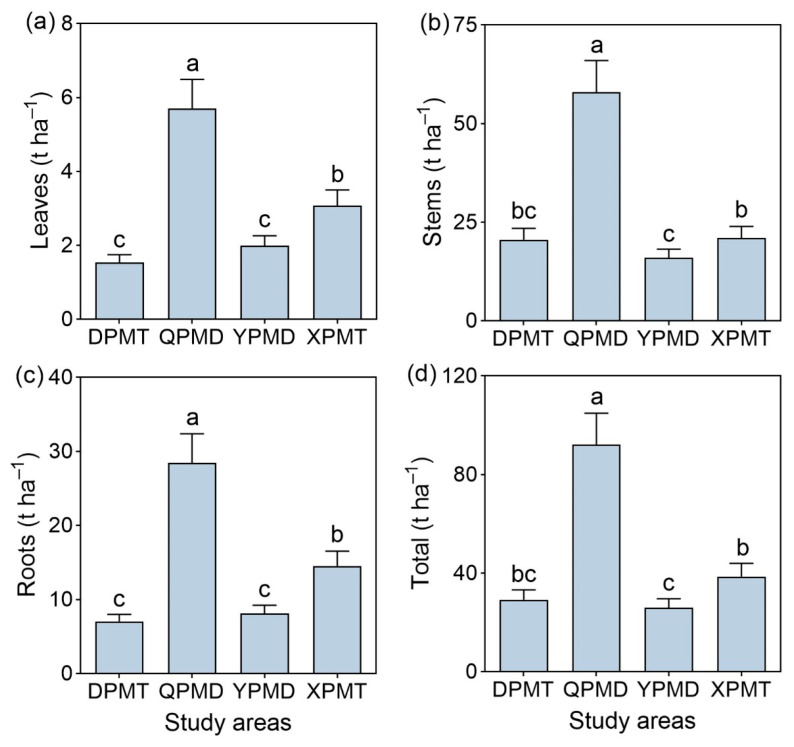
Variation in plant biomass across the studied mangrove areas of Hainan (t ha^−1^). (**a**) Leaf biomass; (**b**) Stem biomass; (**c**) Root biomass; and (**d**) Total biomass, calculated as the sum of leaves, stems, and roots. Different lowercase letters indicate significant differences among regions (DPMD, QPMD, YBMD, and XPMD), as determined by one-way ANOVA followed by Tukey’s HSD test (*p* < 0.05), as determined by one-way ANOVA followed by Tukey’s HSD test.

Plant biomass exhibited clear and statistically significant spatial variability among the four districts (Figure 2). Leaf biomass ranged from 1.5 t ha^−1^ (DPMD) to 5.5 t ha^−1^ (QPMD). QPMD showed the highest leaf biomass, significantly exceeding the overall average across all sampling plots (*p* < 0.05), while DPMD and YBMD had significantly lower values. Stem biomass ranged from approximately 15 t ha^−1^ (DPMD) to 60 t ha^−1^ (QPMD). QPMD recorded the highest stem biomass, significantly greater than the provincial mean (*p* < 0.05). Root biomass followed a similar pattern, with QPMD exceeding 30 t ha^−1^, significantly higher than all other districts (*p* < 0.05). Total biomass ranged from approximately 26 t ha^−1^ (YBMD) to over 92 t ha^−1^ (QPMD). QPMD had the highest total biomass, significantly surpassing the overall average across all sampling plots and all other regions (*p* < 0.05). DPMD exhibited significantly lower total biomass, while XPMD showed values statistically similar to the average.

Plant carbon storage exhibited significant spatial variability among the four mangrove districts, which was generally consistent with, but not identical to, the spatial pattern of total biomass. QPMD stored the highest amount of carbon, significantly exceeding all other regions (*p* < 0.01), followed by XPMD > DPMD > YBMD.

### 2.3. Biomass Allocation Patterns and Ratios

**Figure 3:**
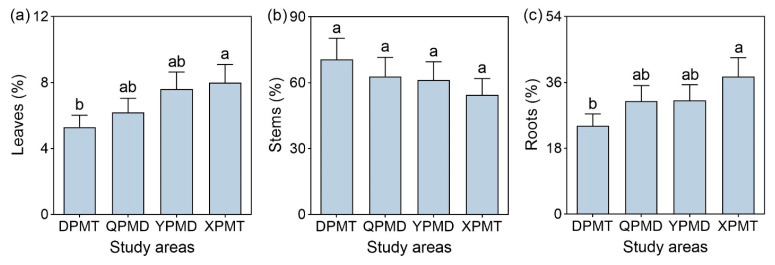
Proportion of biomass contributed by different plant organs to total plant biomass across the studied mangrove areas of Hainan Island. (**a**) Leaves; (**b**) Stems; and (**c**) Roots. Different lowercase letters indicate significant differences among regions (DPMD, QPMD, YBMD, and XPMD), as determined by one-way ANOVA followed by Tukey’s HSD test (*p* < 0.05), as determined by one-way ANOVA followed by Tukey’s HSD test.

The relative contributions of leaves, stems, and roots to total plant biomass exhibited moderate spatial variation (Figure 3). Leaf biomass accounted for approximately 5–10% of total biomass across districts. The highest leaf proportion was observed in XPMD, significantly greater than in DPMD (*p* < 0.05). Stems represented the largest share of biomass in all districts, contributing approximately 60–80% of total biomass. DPMD had the highest stem proportion, significantly exceeding XPMD (*p* < 0.05). Root biomass comprised approximately 20–40% of total biomass. XPMD exhibited the highest root percentage, significantly greater than DPMD (*p* < 0.05), which had the lowest root allocation.

**Figure 4:**
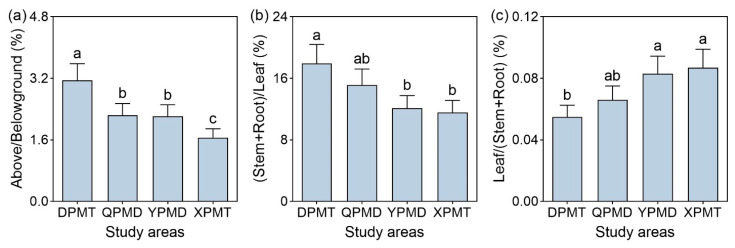
Ratios of biomass among different plant organs across the studied mangrove areas of Hainan. (**a**) Aboveground biomass/Belowground biomass ratio, (**b**) Stem + Root biomass/leaf biomass ratio, (**c**) Leaf biomass/(Stem + Root) biomass ratio. Different lowercase letters indicate significant differences among regions (DPMD, QPMD, YBMD, and XPMD), as determined by one-way ANOVA followed by Tukey’s HSD test (*p* < 0.05).

The aboveground-to-belowground biomass ratio (AGB/BGB) ranged from approximately 1.5 to 3.5 (Figure 4a). DPMD showed the highest AGB/BGB ratio, significantly exceeding all other districts (*p* < 0.05). XPMD had the lowest ratio, significantly lower than the overall average across all sampling plots. The (stem+root)/leaf ratio (Figure 4b) followed a similar trend. DPMD presented the highest ratio, significantly greater than YBMD and XPMD (*p* < 0.05). The leaf/(stem+root) ratio (Figure 4c) showed the inverse pattern. XPMD had the highest ratio, significantly higher than DPMD (*p* < 0.05).

## 3. Discussion 

### 3.1. Species Dominance, Diversity, and Regional Variability

The consistent dominance of *R. stylosa* across all districts reflects its broad ecological tolerance and structural importance in tropical Asian mangroves [3]. The widespread occurrence of this species across contrasting environmental settings indicates its high ecological plasticity and competitive ability under a range of hydrological and edaphic conditions. The high IVI values observed in all four districts suggest that the functional traits of *R. stylosa* may contribute to its competitive advantage and widespread distribution under the diverse environmental conditions of Hainan mangroves. This species possesses several key functional traits that facilitate its success across diverse environmental conditions: (1) a complex stilt root system providing mechanical stability in soft sediments and facilitating gas exchange in anoxic soils, (2) salt excretion through specialized glands on leaves, allowing tolerance of salinities up to 35–40 ppt, (3) viviparous seedling production, which enhances establishment success in intertidal environments, and (4) relatively fast growth rates compared to other Rhizophora species [38].

The district-level variation in IVI patterns provides insight into how local environmental conditions shape community assembly. In DPMD, the co-dominance of *A. corniculatum*, *K. obovata* and *R. stylosa* indicates a mixed-species community where competitive exclusion is incomplete, allowing multiple species to coexist. Similar multi-species assemblages have been reported in relatively sheltered mangrove environments in southern China and Southeast Asia, where moderate freshwater inputs and heterogeneous tidal gradients create diverse ecological niches [39]. This pattern aligns with the intermediate disturbance hypothesis and with previous studies showing that Aegiceras and Kandelia species occupy distinct zones within the intertidal gradient [40]. Their co-occurrence suggests that spatial heterogeneity in micro-elevation, tidal inundation frequency, and sediment characteristics creates multiple niches that support different species.

The strong dominance of *S. apetala* in QPMD suggests a community undergoing early successional development or recovery following disturbance. *S. apetala* is a fast-growing pioneer species that typically colonizes open mudflats and disturbed sites [41]. Its high IVI in QPMD and absence from other districts suggests that QPMD may contain extensive areas of early-successional or regenerating forest, possibly following historical disturbance such as aquaculture conversion [42]. The low richness and dominance by pioneer species indicate that restoration should prioritize increasing native species heterogeneity and facilitating late-successional species recruitment rather than relying solely on fast-growing colonizers. The very low species richness in QPMD (S = 3) indicates a simplified community structure, which may be associated with historical disturbance, environmental filtering, or limited species recruitment.

In YBMD, the balanced co-dominance of *R. stylosa*, *L. racemosa* and *K. obovata* suggests a more mature, structurally complex forest. Comparable patterns have been observed in relatively stable mangrove forests of Southeast Asia, where coexistence among multiple canopy species contributes to higher community stability and functional diversity [43,44]. In XPMD, the extreme dominance of *R. stylosa* suggests very strong environmental filtering favoring this species while excluding others, as the northwestern coast receives lower precipitation and experiences higher evaporation, leading to higher and more variable soil salinities [45].

The pronounced spatial variation in species diversity indices may be associated with differences in environmental conditions, including salinity, hydrology, sediment characteristics, and disturbance history. Salinity is widely recognized as a primary determinant of mangrove species distribution and diversity, as it imposes physiological constraints on water uptake, carbon assimilation, and growth [46]. DPMD, with the highest Shannon diversity and intermediate value of Pielou evenness, has previously been characterized by greater freshwater influence, which may provide suitable conditions for the coexistence of multiple mangrove species [47]. This pattern is consistent with observations from other Asian mangrove systems, where reduced salinity stress and greater habitat heterogeneity generally support higher species richness and evenness [48].

YBMD, with intermediate diversity (H′ = 1.32), may experience somewhat higher salinity (25–35 ppt), reducing the pool of species but still allowing co-dominance of several salt-tolerant species. XPMD exhibits low diversity (H′ = 0.90) and low evenness, suggesting strong dominance by a limited number of species, potentially associated with environmental filtering and local site conditions [40]. QPMD, despite having the lowest species richness, is not necessarily the most saline district; rather, its low diversity likely reflects successional stage and disturbance history. This distinction highlights that low diversity in mangroves may result from both environmental filtering and anthropogenic disturbance, and therefore management strategies should consider the underlying ecological context rather than diversity metrics alone.

Our results highlight the importance of using multiple diversity indices to evaluate community structure comprehensively, as reliance solely on species richness would have missed critical differences among districts [49]. For example, DPMD exhibited relatively high evenness and Shannon diversity, indicating balanced species abundance distribution, whereas XPMD showed lower diversity primarily because of strong dominance by a single species. Similarly, YPMD demonstrated that low richness may reflect successional processes rather than solely environmental stress.

### 3.2. Biomass Production, Vegetation Biomass Carbon Storage, and Management Implications

The pronounced spatial differences in mangrove biomass across districts observed in this study reflect underlying variation in environmental conditions, species composition, and disturbance histories. Rather than being determined by a single environmental factor, biomass accumulation in mangroves results from interactions among species traits, stand structure, hydrological conditions, sediment characteristics, and disturbance regimes. The consistently higher biomass in QPMD may be associated with a combination of favorable site conditions, community composition, and the presence of fast-growing *S. apetala*. Previous studies have shown that freshwater availability, sediment characteristics, and disturbance history can strongly influence mangrove biomass accumulation [1,9]. The dominance of *S. apetala* and *R. stylosa* in QPMD suggests that species composition was an important contributor to the observed biomass differences among districts. The contribution of *S. apetala* is particularly important because this pioneer species generally exhibits rapid growth and high productivity during early forest development, allowing disturbed or newly established mangrove stands to accumulate biomass quickly [50]. The elevated root biomass in QPMD indicates greater belowground biomass allocation, which may contribute to differences in ecosystem carbon dynamics [51].

The significantly lower biomass in YBMD and DPMD may be associated with differences in species composition, stand structure, environmental conditions, or historical disturbance. In DPMD, the dominance of *A. corniculatum* and *K. obovata*, which generally have smaller maximum stature than Rhizophora and Sonneratia species, may inherently limit biomass accumulation [52]. In YBMD, differences in site conditions and stand characteristics may contribute to the lower biomass observed [53]. Biomass in XPMD was intermediate, with elevated leaf and root proportions suggesting adaptation to stress [54,55]. Across all districts, stems represented the largest proportion of total biomass, consistent with global mangrove allometric patterns [51], suggesting that fundamental allometric scaling relationships are relatively conserved across mangrove species and environments [56].

Vegetation biomass and plant carbon storage varied substantially among districts and closely followed patterns of biomass accumulation. This strong correspondence indicates that stand biomass was the primary determinant of vegetation carbon storage in these mangrove forests. These vegetation biomass carbon values are within the range previously reported for Chinese mangroves [22,27,28]. However, these estimates represent only the vegetation biomass carbon pool and do not include soil organic carbon, which represents a major component of total mangrove ecosystem carbon stocks. In mangrove ecosystems, soil carbon typically accounts for 50–90% of total ecosystem carbon [5,9,22]. Therefore, future studies incorporating soil carbon measurements are required to quantify complete ecosystem carbon stocks in these mangrove forests. Although some districts with higher plant diversity values also exhibited relatively higher biomass and vegetation biomass carbon storage, these patterns represent spatial associations rather than evidence that plant diversity alone determines ecosystem carbon accumulation. Instead, biomass production and carbon storage likely emerge from the combined effects of species composition, functional traits, environmental conditions, and stand development history. Similar findings from Asian mangrove systems indicate that carbon storage differences are strongly influenced by forest age, dominant species, hydrological conditions, and disturbance history rather than diversity alone [1,57].

The strong spatial variability has direct management implications. Because the four regions represent distinct ecological conditions, conservation and restoration strategies should be tailored to local community characteristics rather than applying a uniform management approach. QPMD should be prioritized for protection due to its high carbon biomass and vegetation carbon storage, while management should also focus on maintaining species heterogeneity and promoting the transition from pioneer-dominated stands toward more structurally complex communities. DPMD should be managed to maintain its high plant diversity and balanced species composition, with emphasis on conserving existing mixed-species assemblages and protecting habitats that support multiple native mangrove species. XPMD characterized by a relatively balanced co-dominance structure, should prioritize maintaining habitat stability and preventing disturbances that could disrupt species coexistence and reduce ecosystem resilience. YBMD, where strong dominance by *R. stylosa* occurs under comparatively stressful environmental conditions, may benefit from restoration strategies that enhance habitat heterogeneity and facilitate the recruitment of additional native species, rather than replacing the dominant native species. These region-specific approaches are consistent with broader mangrove restoration recommendations in Asia, which emphasize ecological suitability, natural regeneration, and restoration of ecosystem processes rather than single-species planting programs [58,59]. Region-specific strategies are necessary because the substantial ecological heterogeneity observed among mangrove areas indicates that uniform management approaches may not effectively address local conservation and restoration needs. Management strategies should consider differences in species composition, community structure, biomass distribution, and carbon storage potential among regions to optimize mangrove conservation and restoration outcomes.

### 3.3. Biomass Allocation Patterns and Functional Strategies

The proportional allocation of biomass among leaves, stems, and roots provides insight into how these forests balance competing demands [60,61]. Stems consistently dominated (60–80%), reflecting the structural investment required to support canopy development and withstand hydrodynamic forces in intertidal environments [1,9]. This dominance of stem biomass is consistent with global patterns reported for mangrove ecosystems, where long-lived woody tissues represent the primary carbon storage component and provide mechanical stability under tidal disturbance. Variation in leaf and root allocation observed among the four districts reveals district-level differences in physiological strategies and suggests contrasting adaptation patterns under different environmental conditions.

The higher leaf percentage in XPMD compared to DPMD may indicate greater investment in photosynthetic tissues, potentially enhancing carbon acquisition under conditions where light availability is not limiting but environmental stress may constrain growth [62]. The higher root proportion in XPMD suggests adaptation to the relatively stressful environmental conditions of northwestern Hainan, where higher salinity variability, lower precipitation, and greater evaporative demand may increase the importance of belowground resource acquisition and water regulation [63,64]. Similar increases in root allocation have been reported in mangroves exposed to salinity stress, nutrient limitation, or unstable sediments, reflecting a shift toward belowground investment to maintain physiological functioning.

The AGB/BGB ratio ranged among districts, reflecting contrasting functional strategies. A higher AGB/BGB ratio indicates greater investment in aboveground structures, whereas a lower ratio reflects relatively greater allocation to belowground components. DPMD’s high ratio suggests greater allocation toward aboveground biomass, which may be associated with differences in stand structure and local environmental conditions. XPMD’s low ratio suggests sediment instability, nutrient limitation, or osmotic stress that favors root development and belowground resource acquisition [65,66]. The higher root investment observed in XPMD may represent an adaptive response to the relatively stressful environmental conditions characteristic of the northwestern coast of Hainan. From an ecosystem perspective, these allocation differences may influence long-term carbon dynamics because belowground biomass contributes directly to soil organic matter formation and carbon persistence through root turnover and rhizodeposition [64]. Therefore, differences in biomass allocation among districts highlight that mangrove carbon storage is regulated not only by total biomass accumulation but also by species-specific functional strategies and environmental adaptation.

### 3.4. Limitations and Future Directions

This study has several limitations. First and most significantly, soil carbon was not measured, despite soil representing the largest carbon pool in most mangroves [1,5]. Future studies should include systematic soil sampling to quantify total ecosystem carbon stocks. Second, the data represent a single temporal snapshot (2023) and do not capture interannual variability. Long-term monitoring across multiple years and disturbance gradients will be necessary to evaluate temporal dynamics and ecosystem responses to environmental change. Third, no direct measurements of environmental variables (salinity, nutrients, and sediment properties) were collected across all plots. Future studies should integrate detailed environmental measurements with floristic and biomass surveys to enable mechanistic analysis of the drivers of spatial variation. Future studies should integrate detailed environmental measurements with floristic surveys and biomass assessments to identify the mechanisms driving spatial variation in mangrove community structure and carbon storage.

In addition, this study primarily focused on characterizing spatial patterns of plant diversity, biomass allocation, and vegetation biomass carbon storage among representative mangrove districts. Although differences in plant diversity characteristics were observed concurrently with variations in biomass and carbon storage, the present dataset does not include quantitative modeling approaches (e.g., correlation analyses, regression models, or structural equation modeling) to directly evaluate causal relationships between plant diversity and vegetation biomass carbon accumulation. Therefore, the observed relationships should be interpreted as spatial associations rather than direct evidence that plant diversity determines biomass production or vegetation biomass carbon storage. Future studies combining long-term plant diversity monitoring, environmental measurements, and quantitative modeling approaches will be essential for disentangling the relative contributions of species composition, environmental filtering, and disturbance history to mangrove ecosystem functioning.

The findings from this study can be applied in several practical contexts: (a) providing a baseline for mangrove carbon accounting frameworks to support climate change mitigation policies such as REDD+; (b) informing the prioritization of areas for ecosystem restoration or conservation based on spatial variation in biomass and structure; (c) supporting coastal protection planning by integrating vegetation data into models of wave attenuation and shoreline stabilization; and (d) serving as ground-based reference data for calibrating remote sensing models (e.g., satellite or drone-based) aimed at mapping mangrove biomass and health across larger scales. Building on the limitations and findings of this study, future research should focus on the following: (1) establishing long-term, multi-site monitoring networks across biogeographic and disturbance gradients to capture interannual and decadal trends; (2) designing integrated assessments that simultaneously measure soil carbon pools, vegetation structure, and environmental drivers (salinity, nutrients, sediment accretion) to enable mechanistic modeling; (3) conducting repeated surveys to quantify the impacts of extreme events (storms, sea-level rise, droughts) and recovery trajectories of disturbed mangroves; (4) fusing high-resolution remote sensing (optical, radar, LiDAR) with systematic ground truthing to improve non-destructive estimates of total ecosystem carbon stocks; and (5) conducting experimental manipulations (field or mesocosm studies) to evaluate causal relationships between environmental stressors and mangrove growth, mortality, biomass allocation, and carbon dynamics. Such integrated observational and experimental approaches will improve understanding of how plant diversity, environmental variability, and climate-related disturbances interact to regulate mangrove ecosystem resilience and carbon sequestration.

## 4. Materials and Methods

### 4.1. Study Sites

**Figure 5:**
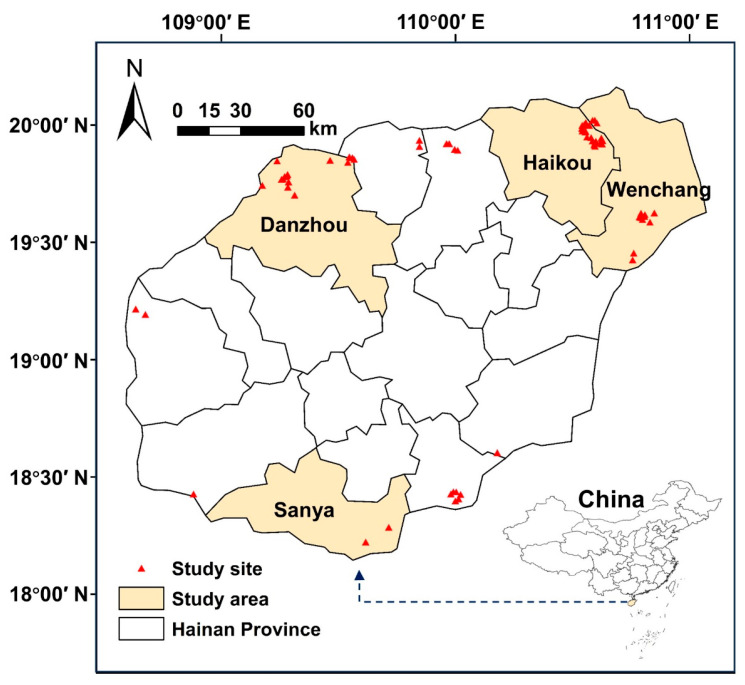
Geographical locations of the study areas in Hainan Province, China, showing the four major mangrove regions based on geographic location and environmental characteristics. Northeastern region: Dongzhai Port mangrove district (DPMD) in Haikou; Eastern region: Qinglan Port mangrove district (QPMD) in Wenchang; Southern region: Yalong Bay mangrove district (YBMD) in Sanya; and Northwestern region: Xinying Port mangrove district (XPMD) in Danzhou.

This study was conducted in representative mangrove forest ecosystems along the coastal harbors and river estuaries of Hainan Province, located in the tropical zone of southern China (108°21′–111°03′ E, 19°20′–20°10′ N) (Figure 5). Hainan is the largest tropical island in China, with a coastline of approximately 1944 km, of which approximately 10% supports mangrove vegetation [26]. The island’s climate is characterized by distinct wet (May–October) and dry (November–April) seasons, with annual precipitation ranging from 1500 mm in the west to over 2500 mm in the east and southeast [25]. Temperature regimes are relatively uniform across the island due to its tropical location, with mean annual temperatures of 22–24 °C in the northern and western regions and 24–26 °C in the southern region [27].

To ensure comprehensive spatial representation, the mangrove forests were stratified into four major ecological districts based on geographic location, geomorphology, and environmental characteristics:

(1) Dongzhai Port (DPMD), Haikou (19°57′ N, 110°34′ E): A Ramsar site with a complex deltaic system fed by the Yanfeng River. Tidal range is 1.5–2.2 m, salinity ranges from 15–32 ppt, and anthropogenic pressure is low due to strict protection since 1986.
(2) Qinglan Port (QPMD), Wenchang (19°33′ N, 110°50′ E): The largest contiguous mangrove area on Hainan, located at the mouth of the Wenchang River with a broad estuarine embayment. Tidal range is 1.2–1.8 m, salinity ranges from 10–30 ppt, and some areas have experienced historical disturbance from aquaculture.
(3) Yalong Bay (YBMD), Sanya (18°13′ N, 109°38′ E): A fringing mangrove system in southern Hainan with a pronounced dry season, elevated salinity (25–35 ppt), small tidal range (0.8–1.2 m), and moderate pressure from urban and tourism development.
(4) Xinying Port (XPMD), Danzhou (19°44′ N, 109°23′ E): Located on the northwestern coast with lower precipitation (1200–1500 mm), higher evaporation, and higher variable salinity (20–35 ppt). Tidal range is 1.5–2.0 m, and the area has experienced intensive historical disturbance from aquaculture and salt ponds.

These four districts encompass broad environmental gradients in precipitation, salinity, tidal hydrology, and disturbance, providing a robust basis for evaluating spatial heterogeneity in mangrove structure, plant diversity, biomass and carbon allocation across Hainan Island. To provide an overall reference for mangrove ecosystems across Hainan Island, mean values were calculated from the four ecological regions (DPMD, QPMD, YBMD, and XPMD). These values represent the general spatial patterns of mangrove vegetation characteristics across the island and were included only for descriptive purposes in the figures. They were not treated as an independent group in statistical analyses. Analyses of variance and comparisons of means were conducted using plot-level data from the four ecological districts, with each district representing a spatial sampling category.

### 4.2. Experimental Design, Field Sampling, and Biomass Estimation

This study employed a stratified spatial sampling design to comprehensively assess biodiversity, vegetation structure, biomass production, and carbon allocation across the mangrove ecosystems of Hainan Province. Fieldwork was conducted throughout the calendar year of 2023 as part of the National Forest-Grass-Wetland Integrated Monitoring Program, a comprehensive national-scale ecological monitoring effort coordinated by the National Forestry and Grassland Administration of China [67]. Although field surveys were conducted during different periods of 2023 due to the requirements of the national monitoring schedule and site accessibility, all plots were surveyed following standardized protocols for vegetation inventory, biomass estimation, and organ-level measurements. Seasonal variations in leaf biomass were not explicitly quantified; however, the consistent sampling procedures and measurement methods helped minimize methodological inconsistencies and ensured the comparability of biomass and carbon storage estimates among the four mangrove districts. A total of 73 circular sample plots were established across the four study districts, with the number of plots proportional to the mangrove area in each district: DPMD (n = 22), QPMD (n = 18), YBMD (n = 15), and XPMD (n = 18). The geographic coordinates of each sampling plot were recorded using a handheld GPS receiver during field surveys to ensure accurate spatial referencing of sampling locations. Each plot had a radius of 40 m, corresponding to an area of approximately 0.5 ha, providing a standardized sampling unit of sufficient size to capture stand-level structural variation while remaining logistically manageable in challenging mangrove terrain [68]. Within each plot, all vascular plant species were identified and recorded to quantify species richness and community composition. Woody vegetation was surveyed using a nested approach: all trees with diameter at breast height (DBH) ≥ 5 cm were measured for DBH and total height, while shrubs and tree seedlings (DBH < 5 cm) were counted and measured for height and canopy dimensions (length and width).

As the study area is located within protected zones, this study did not employ destructive harvest methods to directly measure plant biomass. Instead, we utilized published, species-specific allometric equations developed for mangrove species in southern China (Table 3) to calculate the biomass of leaves, branches/stems, and roots [68]. Individual tree biomass was derived by summing the three component estimates. The allometric equations used in this study were directly adopted from previously published studies rather than developed using the present dataset. Therefore, model performance parameters (e.g., R^2^ and RMSE) were not recalculated in this study. The goodness-of-fit statistics of the original models, where available, are reported in the source publications. The use of established regional allometric models enabled consistent biomass estimation across different mangrove districts while avoiding destructive sampling in protected ecosystems. Total plant biomass within each quadrat was obtained by aggregating the biomass of all trees within the 0.5-ha plot, then scaled to a per-hectare basis (t ha^−1^). Plant carbon storage for each quadrat was calculated by multiplying the plant biomass (t ha^−1^) by the plant carbon content fraction (%). The carbon content values used in this study (Table 4) were based on previously published data from mangrove species in southern China [29,31,32,37,69].

### 4.3. Importance Value and Plany Diversity Indices

To evaluate the ecological significance of plant species within the mangrove communities, the Importance Value Index (IVI) was calculated for each species at both the regional (pooled across all regions) and region-specific levels. The IVI is a composite metric that integrates relative abundance, relative frequency, and relative dominance, providing a comprehensive measure of a species’ contribution to community structure and function [70]. IVI was calculated as:

(1) $$IVI=\frac{\left(RA+RF+RD\right)}{3}$$

where RA, RF, and RD represent the relative density, relative frequency, and relative dominance, respectively, each expressed as a percentage. Density is the number of individuals of a species per unit area, frequency is the proportion of plots in which the species occurs, and dominance is the total basal area of the species per unit area.

In addition to IVI, four standard diversity indices were calculated to quantify species richness, diversity and evenness at the plot level [71], then averaged across districts:

Margalef species richness index (k):

(2) $$K=\frac{S-1}{lnN}$$

Shannon-Wiener diversity index (H′):

(3) $$H^{\prime }=-\sum_{i=1}^{S}P_{i}lnP_{i}$$

Simpson dominance index (D):

(4) $$D=\sum_{i=1}^{S}\frac{n_{i}(n_{i}-1)}{N(N-1)}$$

Pielou evenness index (J):

(5) $$J=\frac{H^{\prime }}{lnS}$$

where, S is the total number of species in a plot, ni is the number of individuals of species i, N is the total number of individuals of all species in the plot, and Pi is the relative proportion of species i to the total number of individuals in the plot. Fisher’s alpha diversity index (α) was also calculated to provide an additional measure of species diversity based on the relationship between species richness and abundance. Fisher’s alpha is derived from the Fisher log-series distribution [72] and was estimated using the following equation:

(6) $$S=\alpha ln\left(1+\frac{N}{\alpha }\right)$$

where S is the total number of species, N is the total number of individuals, and α is Fisher’s alpha diversity index. The value of α was estimated iteratively from the observed species richness and total abundance for each mangrove ecological district.

### 4.4. Data Analysis

All field data were compiled and processed in Microsoft Excel prior to statistical analysis. All statistical analyses were performed in R version 4.3.2, with significance evaluated at α = 0.05. The R packages (v4.4.0) used for statistical analyses included car for Levene’s tests, stats for Shapiro–Wilk tests, one-way ANOVA, and Tukey’s HSD tests, and FSA for Dunn’s tests with Benjamini–Hochberg correction. For each variable of interest (species richness, Fisher’s alpha diversity index, IVI, other plant diversity indices, biomass components, biomass ratios, carbon storage), we first tested the assumptions of normality (using Shapiro–Wilk tests) and homogeneity of variances (using Levene’s tests) across the four districts. When data met parametric assumptions, regional differences were assessed using one-way analysis of variance (ANOVA). When ANOVA indicated significant overall differences (*p* < 0.05), post-hoc pairwise comparisons were conducted using Tukey’s Honestly Significant Difference (HSD) test. When data did not meet parametric assumptions, we used the non-parametric Kruskal–Wallis rank-sum test, followed by Dunn’s test with Benjamini–Hochberg correction for multiple comparisons.

All statistical analyses were performed using plot-level data from the four ecological districts (DPMD, QPMD, YBMD, and XPMD). The ecological districts were considered the spatial comparison units, and no additional provincial-level category or average value was included in the analysis of variance or mean comparisons. Because this study primarily aimed to characterize spatial variation in plant diversity, biomass allocation, and carbon storage among representative mangrove districts, statistical analyses focused on comparisons among districts. Relationships between plant diversity metrics and biomass/carbon storage were interpreted as spatial patterns rather than tested causal relationships. Future studies incorporating correlation analyses, regression models, or structural equation modeling are needed to quantitatively evaluate the mechanisms linking plant diversity and ecosystem carbon storage.

## 5. Conclusions

This study presents a comprehensive assessment of spatial variation in mangrove plant diversity, biomass allocation, and vegetation biomass carbon storage across major mangrove ecosystems in Hainan, China. The findings demonstrate that mangrove communities exhibit strong spatial heterogeneity, with community composition and structural characteristics differing among ecological districts. These differences indicate that mangrove ecosystem patterns are shaped by the combined effects of species-specific traits, environmental conditions, and disturbance history rather than by a single controlling factor. Our results support the conclusion that species composition and biomass allocation strategies are critical determinants of vegetation biomass accumulation and carbon storage. Dominant species and their functional characteristics influence forest structure, while variations in aboveground and belowground biomass allocation reflect regional adaptation to contrasting environmental conditions. However, the observed relationships between plant diversity and vegetation biomass carbon storage represent spatial associations rather than direct causal effects, highlighting the need for integrated approaches that consider multiple ecological drivers. The study further demonstrates that effective mangrove conservation and restoration require region-specific strategies based on local ecological characteristics. Management priorities should integrate biodiversity conservation, maintenance of forest structural complexity, and protection of high-carbon ecosystems. These findings provide a scientific basis for improving mangrove management and supporting blue carbon initiatives in subtropical coastal regions. Future research should incorporate soil carbon measurements, long-term monitoring, and comprehensive assessments of environmental drivers to improve understanding of mangrove ecosystem carbon dynamics and resilience under ongoing environmental change.

## Figures and Tables

**Table 1:** Species composition and quantitative features across all study regions.

Plant Species	Family	Classification	Relative Density (%)	Relative Frequency (%)	Relative Dominance (%)	IVI
*R. stylosa*	Rhizophoraceae	Native true mangroves	50.44	37.70	37.36	41.83
*S. apetala*	Lythraceae	Introduced mangroves	6.74	9.84	18.30	11.63
*K. obovata*	Rhizophoraceae	Native true mangroves	10.35	18.03	3.74	10.71
*A. corniculatum*	Primulaceae	Native true mangroves	8.76	16.39	5.13	10.09
*L. racemosa*	Combretaceae	Native true mangroves	10.73	4.92	7.16	7.60
*H. tiliaceus*	Malvaceae	Mangrove associate	0.61	0.82	11.81	4.41
*L. racemosa*	Combretaceae	Introduced mangroves	4.11	1.64	6.18	3.98
*R. apiculata*	Rhizophoraceae	Native true mangroves	2.63	1.64	5.08	3.12
*A. marina*	Acanthaceae	Native true mangroves	1.48	4.10	0.64	2.08
*B. sexangula*	Rhizophoraceae	Native true mangroves	2.63	2.46	1.09	2.06
*S. caseolaris*	Lythraceae	Native true mangroves	1.14	1.64	1.34	1.37
*B. gymnorrhiza*	Rhizophoraceae	Native true mangroves	0.38	0.82	2.18	1.13
Total			100.00	100.00	100.00	100.00

**Table 2:** Variation of Importance Value Index (IVI, %) of mangrove species across the four districts in Hainan of China.

Species	DPMD (%)	QPMD (%)	YBMD (%)	XPMD (%)
*R. stylosa*	19.77	39.96	35.74	58.75
*S. apetala*	–	44.67	–	–
*K. obovata*	21.84	–	26.46	2.06
*A. corniculatum*	37.49	–	–	3.87
*L. racemosa*	–	15.37	–	11.62
*L. racemosa*	–	–	26.70	–
*H. tiliaceus*	–	–	–	14.25
*R. apiculata*	–	–	–	9.45
*A. marina*	–	–	11.10	–
*B. sexangula*	9.16	–	–	–
*S. caseolaris*	6.17	–	–	–
*B. gymnorrhiza*	5.57	–	–	–

‘–’ indicates species not recorded.

**Table 3:** Biomass regression equations for mangrove plants in the study site [67,68].

Plant Species	Equations
*Rhizophora stylosa*	W_L_ = 0.055(D)^1.687^ W_S_ = 0.085(D^2^H)^0.891^ W_R_ = 0.295(D)^1.575^
*Lumnitzera racemosa*	
*Hibiscus tiliaceus*	
*Bruguiera gymnorrhiza*	
*Sonneratia apetala*	W_L_ = 0.003(D)^2.556^ W_S_ = 0.028(D^2^H)^0.981^ W_R_ = 0.013(D)^2.685^
*Sonneratia caseolaris*	
*Kandelia obovata*	W_L_ = 0.018(D)^1.567^ W_S_ = 0.08 (D^2^H)^0.797^ W_R_ = 0.066(D)^1.847^
*Aegiceras corniculatum*	
*Rhizophora apiculata*	
*Bruguiera sexangula*	
*Bruguiera sexangula*	

W_L_: leave biomass, W_S_: stem biomass, W_R_: root biomass, D: diameter at breast height (cm), H: tree height (m).

**Table 4:** Carbon concentration (%) of mangrove plant species in the study area.

Tree Species	Carbon Concentration (%)	References
*Avicennia marina*	41.3	[29,37]
*Rhizophora stylosa*	43.2	[37]
*Lumnitzera racemosa*	44.3	[37,69]
*Hibiscus tiliaceus*	46.2	[31]
*Bruguiera gymnorrhiza*	44.3	[37,69]
*Sonneratia apetala*	43.6	[29,31]
*Sonneratia caseolaris*	43.0	[29,31]
*Kandelia obovata*	41.3	[29,31]
*Aegiceras corniculatum*	42.9	[31,32]
*Rhizophora apiculata*	48.3	[31]
*Bruguiera sexangula*	49.0	[31]
*Laguncularia racemosa*	47.2	[31]

## Data Availability

The original contributions presented in this study are included in the article. Further inquiries can be directed to the corresponding authors.
